# Characterization of the immune response in ganglia after primary simian varicella virus infection

**DOI:** 10.1007/s13365-015-0408-1

**Published:** 2015-12-16

**Authors:** Werner J. D. Ouwendijk, Sarah Getu, Ravi Mahalingam, Don Gilden, Albert D. M. E. Osterhaus, Georges M. G. M. Verjans

**Affiliations:** Department of Viroscience, Erasmus MC, ‘s-Gravendijkwal 230, 3015 CE Rotterdam, The Netherlands; Department of Neurology, University of Colorado School of Medicine, Aurora, CO USA; Research Center for Emerging Infections and Zoonoses, University of Veterinary Medicine Hannover, Hannover, Germany

**Keywords:** Simian varicella virus, Varicella-zoster virus, Sensory ganglia, Immune response, Primary infection

## Abstract

Primary simian varicella virus (SVV) infection in non-human primates causes varicella, after which the virus becomes latent in ganglionic neurons and reactivates to cause zoster. The host response in ganglia during establishment of latency is ill-defined. Ganglia from five African green monkeys (AGMs) obtained at 9, 13, and 20 days post-intratracheal SVV inoculation (dpi) were analyzed by ex vivo flow cytometry, immunohistochemistry, and in situ hybridization. Ganglia at 13 and 20 dpi exhibited mild inflammation. Immune infiltrates consisted mostly of CD8^dim^ and CD8^bright^ memory T cells, some of which expressed granzyme B, and fewer CD11c^+^ and CD68^+^ cells. Chemoattractant CXCL10 transcripts were expressed in neurons and infiltrating inflammatory cells but did not co-localize with SVV open reading frame 63 (ORF63) RNA expression. Satellite glial cells expressed increased levels of activation markers CD68 and MHC class II at 13 and 20 dpi compared to those at 9 dpi. Overall, local immune responses emerged as viral DNA load in ganglia declined, suggesting that intra-ganglionic immunity contributes to restricting SVV replication.

## Introduction

Varicella-zoster virus (VZV) is a neurotropic human alphaherpesvirus. Primary infection causes varicella (chicken pox), after which VZV becomes latent in ganglionic neurons; reactivation leads to zoster (shingles) (Arvin and Gilden 2013). Virus-specific immunity is essential for uncomplicated recovery from varicella and zoster (Arvin and Gilden 2013). While the magnitude of systemic VZV-specific T cell responses is known to be inversely correlated with the severity of varicella and zoster in immunocompetent individuals (Arvin et al. 1986; Malavige et al. 2008; Weinberg et al. 2009), VZV-specific immune responses in infected tissues have not been studied in detail (Vukmanovic-Stejic et al. 2013, 2015; Zak-Prelich et al. 2003). Zoster is associated with profound inflammation of ganglia, mainly involving infiltrating T cells that are likely to inhibit local VZV replication and spread (Gowrishankar et al. 2010; Steain et al. 2014). However, the immune response in human ganglia after primary VZV infection has not been studied in detail (Berg et al. 1969; Cheatham et al. 1956; Nagashima et al. 1975).

Due to the highly restricted host tropism of VZV, virus and host factors involved in VZV disease cannot be studied in animal models (Arvin and Gilden 2013). Simian varicella virus (SVV) infection of non-human primates parallels VZV infection in humans (Mahalingam and Gilden 2007; Ouwendijk and Verjans 2015). Primary SVV infection causes varicella, after which virus becomes latent in ganglionic neurons along the entire neuraxis and reactivates to cause zoster (Dueland et al. 1992; Kennedy et al. 2004; Mahalingam et al. 1991, 2007). As in human VZV infection, SVV-specific adaptive immune responses are pivotal in limiting disease severity of both primary and recurrent infections, and virus reactivation induces a transient influx of non-cytolytic T cells in ganglia (Haberthur et al. 2011; Kolappaswamy et al. 2007; Ouwendijk et al. 2013a). Furthermore, depletion of CD4^+^ T cells, but not B cells or CD8^+^ T cells, prevents establishment of SVV latency, suggesting the involvement of intra-ganglionic immune responses (Meyer et al. 2013). Here, we aimed to characterize the immune response in ganglia after primary SVV infection of African green monkeys (AGMs) using both wild-type SVV (SVV-wt) and SVV-expressing enhanced green fluorescent protein (SVV-EGFP).

## Materials and methods

### Animal study design

Cells and tissues were collected from AGM intratracheally inoculated with the wild-type deltaherpesvirus strain of SVV (SVV-wt) (*n* = 2) or a recombinant virus, based on the deltaherpesvirus strain, that expresses enhanced green fluorescent protein in infected cells (SVV-EGFP) (*n* = 3) and euthanized at 9 (*n* = 2), 13 (*n* = 2), and 20 days post-infection (dpi) (*n* = 1), as described (Ouwendijk et al. 2013b) (Table 1). SVV-EGFP was created by inserting a Rous sarcoma virus promoter-EGFP gene cassette between the putative polyadenylation site of SVV open reading frame 66 (ORF66) and the putative TATA box for ORF67, resulting in ectopic expression of EGFP in infected cells (Mahalingam et al. 1998). AGMs were housed, and experiments were performed in compliance with European guidelines (EU Directive on Animal Testing 86/609/EEC) and Dutch legislation. The protocol was approved by the independent animal experimentation ethical review committee DCC (Driebergen, the Netherlands; Erasmus MC permit number EMC2374).

**Table 1:** Characteristics of tissues used to analyze the cell types, nucleic acids, and proteins in ganglia of African green monkeys after primary SVV infection

Animal	Virus	Dpi	Level^a^	Symbol^b^	SVV DNA load^c^	Number of ganglia (neurons) analyzed by		
						Flow cytometry	IHC	ISH
269	SVV-wt	9	TG	●	3.90 × 10^3^	–	–	–
			Cervical	■	1.40 × 10^4^	–	9 (2,257)	–
			Thoracic	▲	1.21 × 10^4^	–	5 (1,248)	2 (1,273)
			Lumbar	▼	1.16 × 10^6^	–	–	–
			Sacral	♦	4.65 × 10^4^	–	2 (1,161)	4 (1,973)
294	SVV-EGFP	9	TG	○	4.64 × 10^2^	–	1 (368)	–
			Cervical	□	2.80 × 10^2^	1	–	–
			Thoracic	∆	1.55 × 10^2^	1	3 (325)	–
			Lumbar	∇	1.53 × 10^2^	2	–	–
			Sacral	◊	5.69 × 10^2^	1	3 (566)	3 (1,156)
279	SVV-wt	13	TG	●	1.38 × 10^3^	–	1 (664)	–
			Cervical	■	4.88 × 10^2^	–	5 (2,417)	3 (1,063)
			Thoracic	▲	5.76 × 10^2^	–	6 (625)	–
			Lumbar	▼	3.55 × 10^2^	–	2 (242)	–
			Sacral	♦	2.37 × 10^2^	–	3 (1,598)	4 (1,884)
273	SVV-EGFP	13	TG	○	1.87 × 10^1^	–	1 (368)	–
			Cervical	□	7.22 × 10^0^	1	6 (1,398)	–
			Thoracic	∆	4.68 × 10^0^	–	2 (782)	–
			Lumbar	∇	4.25 × 10^0^	1	4 (1,970)	3 (1,381)
			Sacral	◊	0.38 × 10^0^	1	6 (3,506)	6 (2,470)
283	SVV-EGFP	20	TG	○	5.46 × 10^0^	–	1 (552)	–
			Cervical	□	1.89 × 10^1^	–	4 (1,368)	4 (1,152)
			Thoracic	∆	4.03 × 10^0^	–	–	–
			Lumbar	∇	2.61 × 10^1^	–	3 (872)	–
			Sacral	◊	5.46 × 10^1^	–	5 (2,631)	3 (1,967)
Total number of ganglia (neurons)						8	72 (24,918)	32 (14,319)

*Dpi* days post-infection, *IHC* immunohistochemistry, *ISH* in situ hybridization, – not done

^a^Anatomical level of the neuraxis from which ganglia were obtained. *TG* trigeminal ganglia

**Fig. 1:**
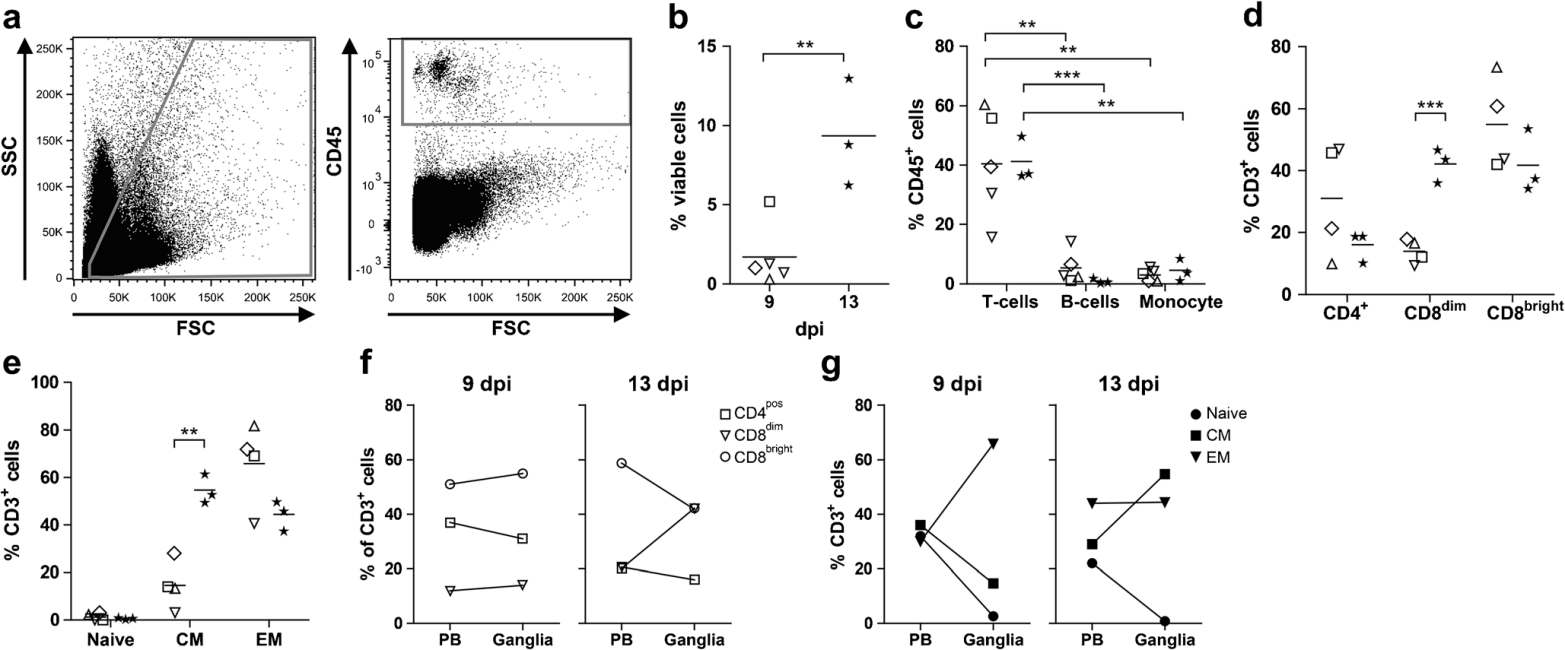
Ex vivo flow cytometry of ganglia after primary SVV infection of African green monkeys. Single-cell suspensions were prepared from eight ganglia obtained from two SVV-EGFP-infected AGMs at 9 days post-infection (dpi) (animal 294; *n* = 5 ganglia) and 13 dpi (animal 273; *n* = 3 ganglia) and analyzed by flow cytometry (Table 1). **a**–**c** Viable cells were selected based on forward scatter (FSC) and sideward scatter (SSC) properties, gated on CD45^+^ leukocytes, and defined as CD3^+^ T cells, CD20^+^HLA-DR^+^ B cells, and CD3^–^CD14^+^HLA-DR^+^ monocytes. **d** T cell subsets were defined as CD4^+^CD8α^−^ “CD4^+^ T cells,” CD4^−^CD8α^dim^ “CD8^dim^ T cells,” and CD4^−^CD8α^bright^ “CD8^bright^ T cells.” **e** T cells were categorized as naïve (CD28^+^CD95^−^), central memory (CM; CD28^+^CD95^+^), or effector memory (EM; CD28^−^CD95^+^) T cells. *Horizontal bars* indicate mean values. *Open symbols* refer to ganglia obtained at 9 dpi and indicate anatomical level of the neuraxis from which ganglia were obtained (Table 1). *Filled stars* refer to individual cervical, lumbar, and sacral ganglia obtained at 13 dpi, from which the original anatomic location of each sample was not annotated in the flow cytometry assay. **f**, **g** Comparison of T cell subsets in paired ganglia and peripheral blood (PB) specimens. **b**–**e** Student’s *t* test was used for statistical analysis. **p < 0.01, ***p < 0.001

**Fig. 2:**
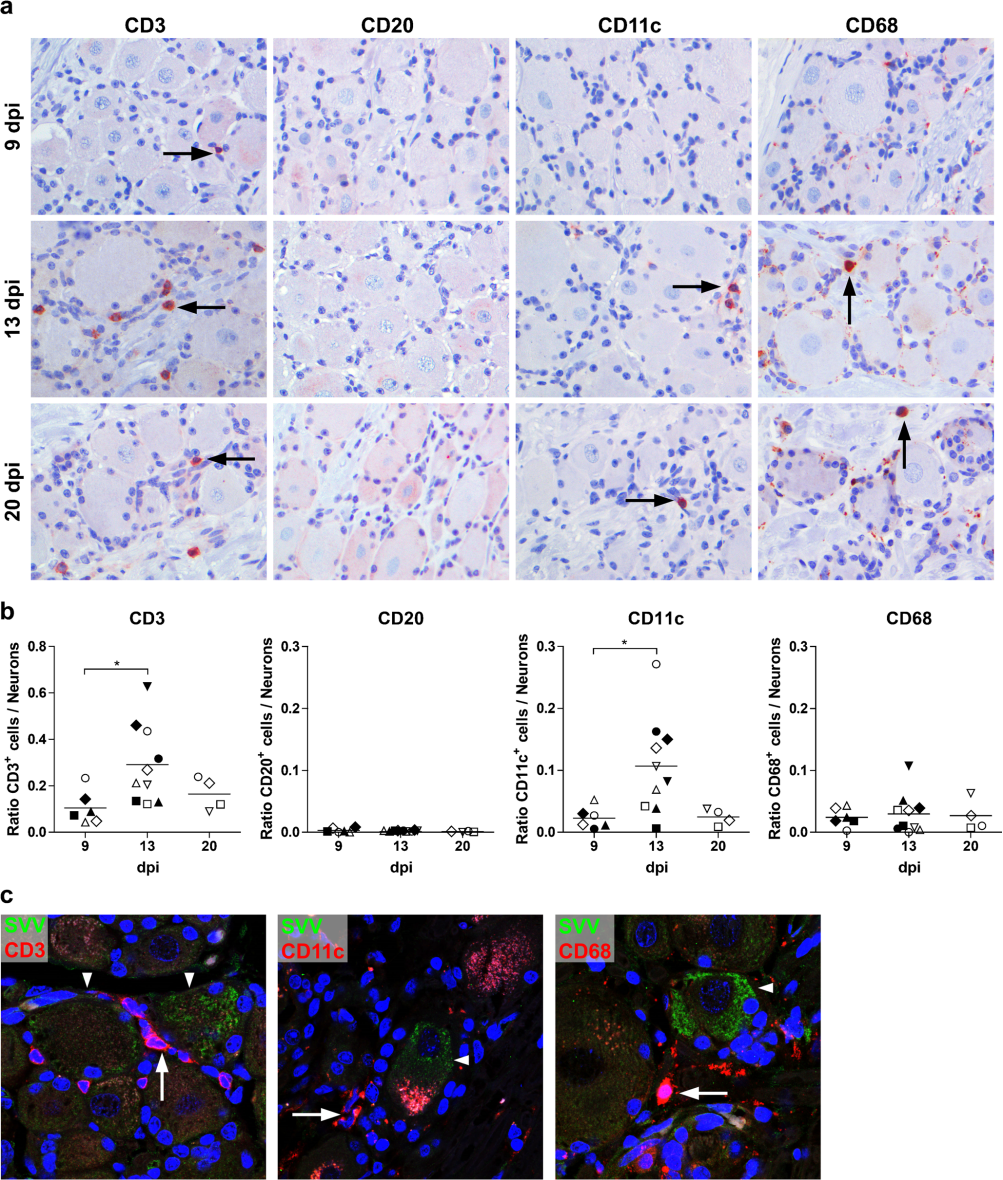
Immunohistochemical analyses of immune cells in ganglia after primary SVV infection of African green monkeys. Immunohistochemical (IHC) staining of ganglia (*n* = 72 ganglia from five animals) at 9, 13, and 20 days post-infection (dpi) for CD3, CD20, CD11c, and CD68. 3-Amino-9-ethylcarbazole (AEC) was used as a substrate (*red*), and nuclei were counterstained with hematoxylin (*blue*). **a** Representative IHC images are shown for 9 dpi [CD3, cervical ganglion from animal 269 (SVV-wt); CD20, CD11c, and CD68, thoracic ganglia from animal 294 (SVV-EGFP)], 13 dpi [CD3, CD20, and CD11c, sacral ganglia from animal 273 (SVV-EGFP); CD68, sacral ganglion from animal 279 (SVV-wt)], and 20 dpi [sacral ganglia from animal 283 (SVV-EGFP)] (see Table 1). Magnification ×400. *Arrows* indicate examples of CD3^+^ cells, CD11c^+^ cells, and CD68^+^ macrophages. **b** Ratio of numbers of CD3^+^, CD20^+^, CD11c^+^, and CD68^+^ cells normalized to the number of neurons in the same tissue section at different dpi. *Horizontal bars* indicate mean values. *Open and filled symbols* refer to ganglia from individual animals infected with SVV-EGFP (*n* = 3) or SVV-wt (*n* = 2), respectively (Table 1). The Student’s *t* test was used for statistical analysis. *p < 0.05. **c** Immunofluorescent double staining for SVV nucleocapsid antigen (*green*) and CD3, CD68, or CD11c (*red*). Representative images of ganglia obtained from animal 279 (SVV-wt) at 13 dpi are shown. Nuclei were counterstained with DAPI. Magnification ×400 and ×1.5 digital zoom. *Arrows* indicate examples of CD3^+^, CD11c^+^, and CD68^+^ cells. *Arrowheads* indicate SVV^+^ neurons

**Fig. 3:**
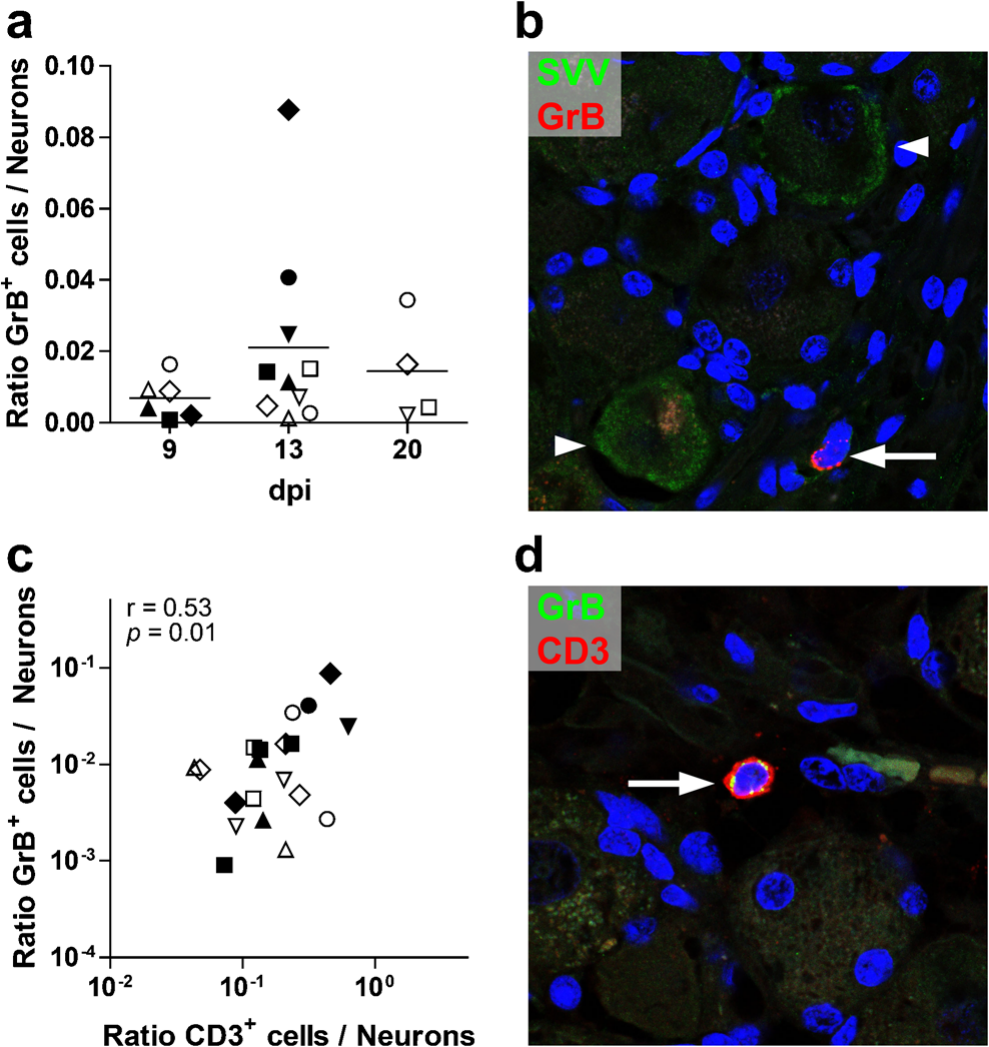
Detection of granzyme B^+^ cells in ganglia after primary SVV infection of African green monkeys. **a** Ratio of numbers of granzyme B (GrB)^+^ cells normalized to the number of neurons in the same tissue section (*n* = 72 ganglia from five animals; see Table 1) at different days post-inoculation (dpi). *Horizontal bars* indicate mean values. **b** Representative confocal microscopy image of a ganglion obtained from a SVV-wt-infected AGM (animal 279) at 13 dpi stained for SVV nucleocapsid antigen (*green*) and GrB (*red*). *Arrow* indicates a GrB^+^ cell, and *arrowheads* indicate SVV^+^ neurons. **c** Scatter plot of the ratio of CD3^+^ cells/neurons versus the ratio of GrB^+^ cells/neurons in consecutive sections. **d** Representative confocal microscopy image of a ganglion obtained from a SVV-wt-infected AGM (animal 279) at 13 dpi stained for GrB (*green*) and CD3 (*red*), showing co-localization of GrB and CD3 (*arrow*). **a**, **c** *Open and filled symbols* refer to ganglia from individual animals infected with SVV-EGFP (*n* = 3) or SVV-wt (*n* = 2), respectively. **b**, **d** Nuclei were counterstained with DAPI. Magnification ×400 and ×2.0 digital zoom. Statistical analysis was performed using the Student’s *t* test (**a**) and Pearson’s correlation test (**c**)

**Fig. 4:**
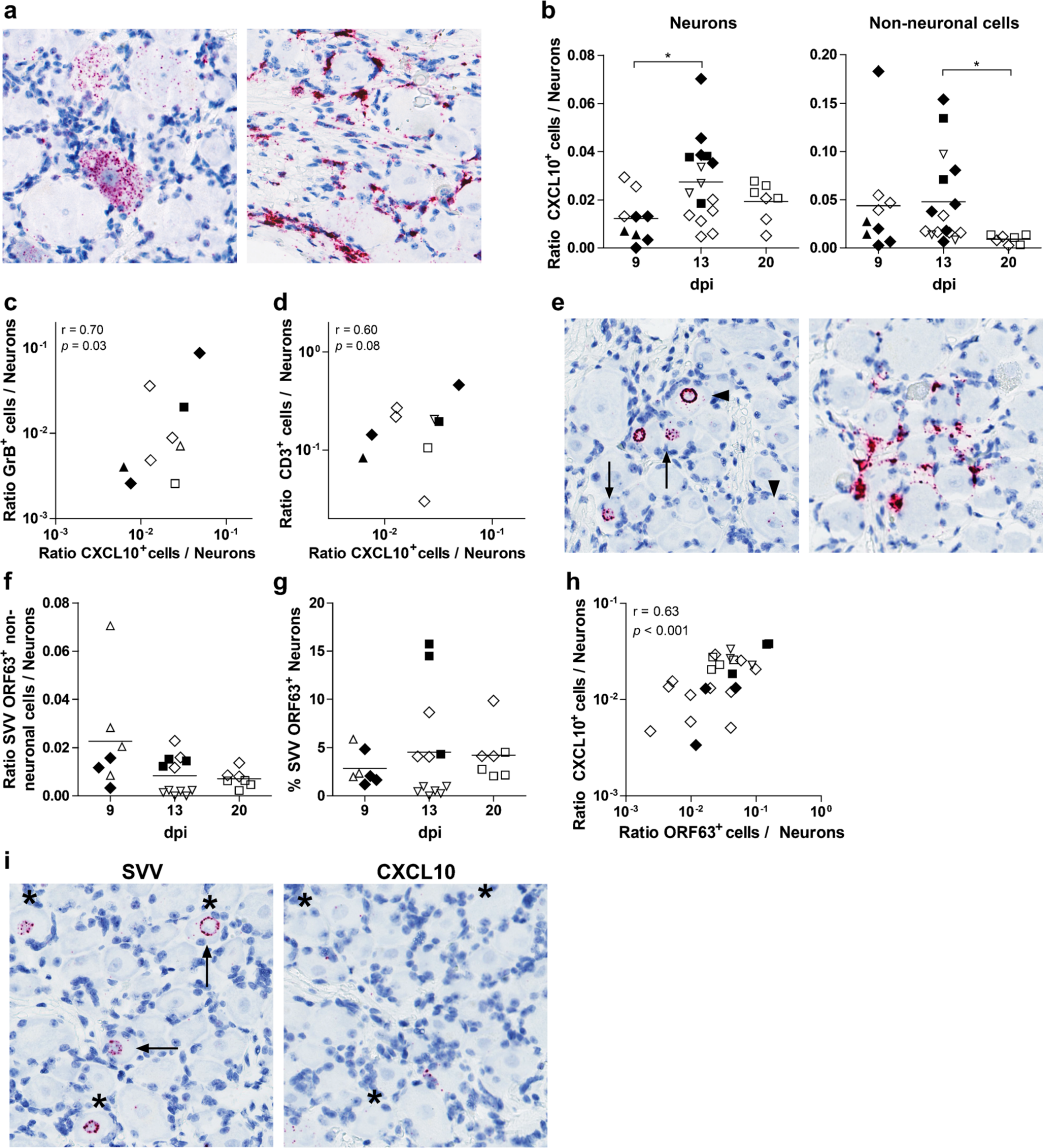
Chemokine CXCL10 expression in ganglia after primary SVV infection of African green monkeys. Ganglia (*n* = 32) obtained from SVV-wt- (*n* = 2) and SVV-EGFP-infected animals (*n* = 3) were analyzed for CXCL10 transcript expression by in situ hybridization (ISH) using FastRed as a substrate (*pink*) and nuclei counterstained with hematoxylin (*blue*) (see Table 1). **a** CXCL10 was detected in both neurons (*left panel*) and non-neuronal cells (*right panel*). *Left panel* sacral ganglion obtained at 13 dpi from a SVV-wt-infected AGM (animal 273). *Right panel* thoracic ganglion obtained at 9 dpi from a SVV-wt-infected AGM (animal 269). Magnification ×400. **b** Ratio of neurons and non-neuronal cells expressing CXCL10 transcripts normalized to the number of neurons in the same section. **c**, **d** Scatter plots of the ratio of CXCL10^+^ neurons/total number of neurons versus the ratio of granzyme B (GrB)^+^ cells/neurons (**c**) and ratio of CD3^+^ cells/neurons (**d**) in the same ganglia. Average values per anatomic level of the neuraxis are plotted. **e** Ganglia were analyzed for SVV ORF63 transcript expression by ISH showing occasional ORF63^+^ non-neuronal cells (*left panel*) and ORF63^+^ neurons (*right panel*). *Left panel* cervical ganglion obtained at 13 dpi from a SVV-wt-infected AGM (animal 279). *Right panel* thoracic ganglion obtained at 9 dpi from a SVV-wt-infected AGM (animal 269). *Arrows* indicate neurons expressing (peri-)nuclear ORF63 transcripts. *Arrowheads* indicate neurons expressing cytoplasmatic and (peri-)nuclear ORF63 transcripts. Magnification ×400. **f** Ratio of SVV ORF63 transcript positive non-neuronal cells normalized to the number of neurons in the same section. **g** Frequency of SVV ORF63 transcript positive neurons. **h** Scatter plot of the ratio of ORF63^+^ neurons/total number of neurons versus the ratio of CXCL10^+^ neurons/total number of neurons in the same ganglia. **i** Representative images of adjacent sections from ganglia obtained at 13 dpi from the SVV-wt-infected animal 279 were stained for SVV ORF63 transcripts and CXCL10 transcripts by ISH. Magnification ×400. *Arrows* indicate examples of SVV ORF63^+^ neurons. *Asterisks* indicate the same neurons in adjacent sections. **b**, **c**, **d**, **f**, **g** *Open and filled symbols* refer to ganglia from individual animals infected with SVV-EGFP or SVV-wt, respectively (Table 1). **b**, **f** Individual ganglia are shown, and *horizontal bars* indicate mean values. Statistical analysis was performed using the Student’s *t* test (**b**, **e**) and Pearson’s correlation test (**c**, **d**, **f**). *p < 0.05

^b^Symbol used in Figs. 1, 2, 3, and 4 to denote anatomical location of ganglia analyzed

^c^Copies of SVV ORF21 DNA per 10^5^ cells, determined as described (Ouwendijk et al. 2013b)

### Flow cytometry

Individual ganglia from two SVV-EGFP-infected AGMs, euthanized at 9 dpi (animal 294; *n* = 5 ganglia) and 13 dpi (animal 273; *n* = 3 ganglia) (Table 1), were dissociated into single-cell suspensions as described (Verjans et al. 2007). Briefly, ganglia were finely diced, enzymatically dissociated using Liberase Blendzyme 3 (0.2 U/ml) (Roche), and filtered through a 70-μm pore size mesh. Single-cell suspensions of individual ganglia and peripheral blood mononuclear cells were stained using fluorochrome-conjugated mouse monoclonal antibodies (mAbs), CD3^APC-Cy7^ (clone SP34-2), CD4^AmCyan^ (L200), CD8^PerCp^ (SK1), CD14^PE^ (M5E2), CD20^PE-Cy7^ (L27), and HLA-DR^PacificBlue^ (L243) (all from BD Biosciences). Single-cell suspensions of ganglia were also stained with a mAb specific for CD45^APC^ (MB4-6D6; Miltenyi Biotec) to distinguish leukocytes from non-leukocytes. T cell subsets were identified using mAbs specific for CD3^APC-Cy7^ (SP34-2), CD4^PacificBlue^ (L200), CD8^AmCyan^ (SK1), CD28^APC^ (28.2), and CD95^PerCp^ (DX2) (all from BD Biosciences). Fluorescence was detected on a FACS Canto II and analyzed using FlowJo software (Tree Star Inc.). Gating strategy for identification of viable cells, leukocytes, and leukocyte subsets is defined in the legend of Fig. 1. On average, 6.5 × 10^2^ and 5.3 × 10^2^ viable leukocytes were analyzed at 9 and 13 dpi.

### Immunohistochemistry

Trigeminal, cervical, thoracic, lumbar, and sacral ganglia were fixed in 4 % paraformaldehyde in phosphate-buffered saline (PBS), paraffin-embedded, and 4-μm sections were analyzed by immunohistochemistry (IHC). We examined multiple ganglia (total *n* = 72 ganglia) from both SVV-wt- and SVV-EGFP-infected AGMs at 9 dpi [animals 269 (SVV-wt) and 294 (SVV-EGFP)], 13 dpi [animals 279 (SVV-wt) and 273 (SVV-EGFP)], and 20 dpi [animal 283 (SVV-EGFP)] (Table 1), and each IHC staining was performed on two to three sections per ganglion at different levels of the tissue. Tissue sections were deparaffinized, rehydrated, and subjected to heat-induced antigen retrieval in citrate buffer (10 mM, pH 6.0); blocked with 10 % normal goat or rabbit serum diluted in PBS; and incubated overnight with primary antibodies diluted in PBS containing 0.1 % BSA (Ouwendijk et al. 2013b). Primary antibodies included mAbs directed against human CD3 (clone F7.2.38; Dako), CD11c (NCL-L-CD11c-563; Novocastra), CD20 (L26; Dako), CD68 (KP1; Dako), granzyme B (GrB-7; Dako), MHC class II (CR3/43; Dako), Ki67 (MIB-1; Dako), rabbit anti-human S100B (EP1576Y; GeneTex), and rabbit polyclonal antibodies directed against SVV nucleocapsid proteins (Dueland et al. 1992). As isotype controls, sections were incubated with the respective mouse isotype antibody (Dako) or rabbit pre-immune serum. For IHC staining, sections were incubated with biotin-conjugated secondary goat anti-mouse or goat anti-rabbit IgG antibodies followed by horseradish peroxidase-conjugated streptavidin (Dako). Staining was visualized using 3-amino-9-ethylcarbazole (AEC; Sigma-Aldrich), and sections were counterstained with hematoxylin (Sigma-Aldrich). For immunofluorescence staining, sections were incubated with secondary Alexa Fluor 488 (AF488)-conjugated goat anti-rabbit Ig, AF594-conjugated goat anti-mouse IgG, AF488-conjugated goat anti-mouse IgG2a, or AF594-conjugated goat anti-mouse IgG1 antibodies and mounted in Prolong Gold Antifade Reagent with 4′,6-diamidino-2-phenylindole (DAPI) (Invitrogen). Sections were analyzed on a Zeiss LSM700 confocal laser scanning microscope (Zeiss) and using ZEN 2010 software (Zeiss) to adjust brightness and contrast.

### In situ hybridization

We examined multiple ganglia (*n* = 32) along the entire neuraxis, from both SVV-wt- and SVV-EGFP-infected AGMs at 9 dpi [animals 269 (SVV-wt) and 294 (SVV-EGFP)], 13 dpi [animals 279 (SVV-wt) and 273 (SVV-EGFP)], and 20 dpi [animal 283 (SVV-EGFP)] (Table 1) by in situ hybridization (ISH), according to the manufacturer’s instructions (Advanced Cell Diagnostics). Briefly, deparaffinized 5-μm tissue sections were incubated with probes against SVV ORF63, CXCL10, ubiquitin C (positive control), and the bacterial DapB gene (negative control) using the RNAscope 2.0 Kit Red (Advanced Cell Diagnostics), as described (Ouwendijk et al. 2013a). Signal was visualized using FastRed as a substrate, and slides were counterstained with hematoxylin and mounted in Ecomount (Biocare Medical). Each ISH staining was performed on one to two sections at different levels of the tissue.

### Quantification of IHC and ISH staining

The numbers of cells expressing CD3, CD11c, CD20, and CD68 [CD68^high^ macrophages, excluding satellite glial cells (SGCs)] protein and CXCL10 transcripts were determined at 200X magnification. The number of granzyme B-expressing cells was determined at 400X magnification. The number of neurons was quantified by scanning the slides using the Nanozoomer 2.0 HT (Hamamatsu) and subsequent analysis by Adobe Photoshop CS6 (Adobe). Overall, 72 ganglia were examined [14.4 ± 4.7 (mean ± standard deviation)] per animal, encompassing 24,918 neurons (Table 1).

### Statistical analyses

Data were assessed for statistical differences using the Student’s *t* test, and correlations were determined using Pearson’s correlation test. Statistical analyses were conducted using GraphPad Prism 5 (GraphPad Software, Inc.), and *p* values <0.05 were considered statistically significant.

## Results

Previously, we inoculated five AGMs intratracheally with SVV-wt (*n* = 2; animals 269 and 279) and SVV-EGFP (*n* = 3; animals 294, 273, and 283) to identify the cell types and route involved in primary SVV infection (Table 1) (Ouwendijk et al. 2013b). Although SVV-EGFP was reported to be not attenuated in vitro and in vivo (Mahalingam et al. 1998, 2001), the virus showed reduced replication and severity of disease compared to SVV-wt in AGM (Ouwendijk et al. 2013b). Nevertheless, the pathogenesis of SVV-EGFP paralleled that of SVV-wt in AGM (Ouwendijk et al. 2013b). Ganglia were obtained at 9 (*n* = 2), 13 (*n* = 2), and 20 dpi (*n* = 1) (Table 1). In AGM infected with SVV-wt or SVV-EGFP, we demonstrated that SVV nucleocapsid antigen was more abundant in ganglia at 9 dpi compared to 13 and 20 dpi (Ouwendijk et al. 2013b). SVV DNA load in ganglia was significantly higher in SVV-wt- compared to SVV-EGFP-infected AGMs, peaking at 9 dpi and decreasing thereafter (Ouwendijk et al. 2013b) (Table 1), as might be expected during the establishment of latency (Messaoudi et al. 2009; Ou et al. 2007; Ouwendijk et al. 2012). Herein, we extended these findings by characterizing the immune response in the ganglia obtained after primary SVV infection.

### Infiltration of CD8^dim^ and CD8^bright^ memory T cells into ganglia after primary SVV infection

To investigate the inflammatory response in ganglia, single-cell suspensions of ganglia from two SVV-EGFP-infected AGMs obtained at 9 dpi (animal 294; *n* = 5 ganglia) and 13 dpi (animal 273; *n* = 3 ganglia) were analyzed by ex vivo flow cytometry (Table 1 and Fig. 1a). Significantly higher frequencies of CD45^+^ leukocytes were found in ganglia at 13 dpi compared to 9 dpi (*p* = 0.006) (Fig. 1b). At both times, CD45^+^ leukocytes were predominantly T cells (Fig. 1c). Unlike humans, AGMs contain three distinct T cell populations identifiable based on differential expressions of CD4 and CD8α, CD4^+^ T cells (CD3^+^CD4^+^CD8α^dim^), CD8^dim^ T cells (CD3^+^CD4^−^CD8α^dim^), and CD8^bright^ T cells (CD3^+^CD4^−^CD8α^high^) (Beaumier et al. 2009). CD4^+^ and CD8^dim^ T cells are interrelated dynamic populations collectively equivalent to human CD4 T cells, while CD8^bright^ T cells correspond to human CD8 T cells (Beaumier et al. 2009). Additionally, non-human primate T cells can be classified as naïve, central memory (CM), and effector memory (EM) populations based on differential expressions of CD28 and CD95 (Beaumier et al. 2009). Whereas CM T cells recirculate between lymphoid tissues and peripheral blood, EM T cells home to peripheral tissues to exert their effector function (Picker et al. 1990; Pitcher et al. 2002). Ganglia obtained at 13 dpi contained significantly higher frequencies of CD8^dim^ (*p* < 0.001) and CM (*p* = 0.002) T cells compared to those obtained at 9 dpi (Fig. 1d, e). Analyses of paired peripheral blood (PB) and ganglionic single-cell suspensions showed similar frequencies of CD4^+^, CD8^dim^, and CD8^bright^ T cells in PB and ganglia at 9 dpi, whereas ganglia were enriched for CD8^dim^ T cells at 13 dpi compared to PB (Fig. 1f). Most ganglionic T cells had an EM phenotype at 9 dpi (Fig. 1g), consistent with their function and findings in human trigeminal ganglia (Verjans et al. 2007). High frequencies of both EM and CM T cells were detected in ganglia at 13 dpi (Fig. 1g). Overall, primary SVV-EGFP infection was associated with increased frequencies of leukocytes, mainly CD8^dim^ and CD8^bright^ T cells of CM and EM phenotypes, in ganglia at 13 dpi compared to 9 dpi.

### Transient increases in CD3^+^ T cells and CD11c^+^ myeloid cells in ganglia

To determine the location of inflammatory cells within ganglia after primary SVV infection, we analyzed ganglia (*n* = 72) obtained at 9, 13, and 20 dpi in AGM infected with either SVV-wt (*n* = 2) or SVV-EGFP (*n* = 3) for the presence of immune cells expressing CD3 (T cells), CD20 (B cells), CD11c (dendritic cells and monocytes/macrophages), and CD68 (macrophages and SGC) by IHC (Table 1). Ganglia from both SVV-wt- and SVV-EGFP-infected AGMs contained similar numbers of immune infiltrates, which peaked at 13 dpi and were composed mostly of CD3^+^ T cells, with lower numbers of CD11c^+^ myeloid cells and CD68^+^ monocytes/macrophages (Fig. 2a; data not shown). These inflammatory cells were dispersed throughout the tissue, but occasionally, clusters of T cells surrounding neurons were observed in both SVV-wt- and SVV-EGFP-infected animals (data not shown). Analysis of the numbers of cells expressing CD3, CD20, CD11c, and CD68 (CD68^high^ macrophages), normalized to the total number of neurons in the same section, showed that primary SVV infection was associated with a transient significant increase in the relative number of CD3^+^ (*p* = 0.02) cells and CD11c^+^ myeloid (*p* = 0.01) cells at 13 dpi compared to 9 dpi (Fig. 2b). Numbers of CD3^+^ and CD11c^+^ cells tended to decrease at 20 dpi. The relative number of CD3^+^ cells and CD11c^+^ cells was not significantly different between SVV-wt- and SVV-EGFP-infected animals at 9 dpi (*p* = 0.92 and *p* = 0.32, respectively) and 13 dpi (*p* = 0.46 and *p* = 0.41, respectively). Notably, occasional CD3^+^, CD11c^+^, and CD68^+^ cells were found adjacent to neurons expressing SVV nucleocapsid antigens in both SVV-wt- and SVV-EGFP-infected AGMs at 13 dpi (Fig. 2c).

### Ganglion-infiltrating T cells express granzyme B

To determine whether T cells infiltrating ganglia were exposed to antigen and are actively involved in local immune responses, we analyzed expression of the cytotoxic T cell marker granzyme B (GrB) in ganglia of AGM infected with either SVV-wt (*n* = 2) or SVV-EGFP (*n* = 3) by IHC. Occasional GrB^+^ cells were detected in ganglia obtained at 9 dpi. Greater numbers of GrB^+^ cells were present in most ganglia obtained at 13 and 20 dpi, although the increase was not statistically significant (*p* = 0.22 and *p* = 0.65, respectively) (Fig. 3a). GrB^+^ cells tended to be more abundant in SVV-wt- compared to SVV-EGFP-infected AGMs at 13 dpi (*p* = 0.07) (Fig. 3a). Some GrB^+^ cells were localized close to SVV nucleocapsid-positive neurons at 13 dpi in both SVV-wt- and SVV-EGFP-infected AGMs (Fig. 3b). The relative numbers of CD3^+^ and GrB^+^ cells in ganglia correlated significantly (*r* = 0.53, *p* = 0.01) (Fig. 3c). Most GrB^+^ cells were also CD3^+^ in adjacent sections (data not shown) and in sections immunofluorescently double stained for CD3 and GrB at 9, 13, and 20 dpi (Fig. 3d), irrespective of the virus used for inoculation. Overall, the ratio of GrB^+^/CD3^+^ cells did not differ at 9 dpi (mean ± standard error of the mean, 0.09 ± 0.36), 13 dpi (0.07 ± 0.20), and 20 dpi (0.07 ± 0.27), suggesting that although more T cells infiltrated ganglia at 13 and 20 dpi, enrichment for antigen-reactive GrB^+^ T cells had not occurred.

### Correlation between numbers of CXCL10^+^ neurons and GrB^+^ T cells

Although earlier studies indicated that chemokine CXCL10 mediates infiltration of T cells into ganglia after VZV and SVV reactivation (Ouwendijk et al. 2013a; Steain et al. 2011), its potential role in primary infection has not been studied. ISH analysis revealed CXCL10 transcripts in both neurons and non-neuronal cells (resident SGC and/or infiltrating leukocytes) in ganglia (*n* = 32) obtained from SVV-wt- (*n* = 2) and SVV-EGFP-infected AGMs (*n* = 3) at 9, 13, and 20 dpi (Fig. 4a and Table 1). Significantly, more neurons expressed CXCL10 transcripts at 13 dpi compared to 9 dpi (*p* = 0.02) (Fig. 4b). Furthermore, more non-neuronal cells expressed CXCL10 transcripts at 13 dpi compared to 20 dpi (*p* = 0.04) (Fig. 4b). The numbers of neuronal and non-neuronal cells expressing CXCL10 were significantly higher in SVV-wt- compared to SVV-EGFP-infected AGMs at 13 dpi (*p* = 0.002 and *p* = 0.02, respectively). Combining data from SVV-wt- and SVV-EGFP-infected animals demonstrated that the number of CXCL10^+^ neurons correlated significantly with the number of GrB^+^ T cells (*r* = 0.70, *p* = 0.03) and tended to correlate with the total number of infiltrating T cells (*r* = 0.60, *p* = 0.08) (Fig. 4c, d). In contrast, the number of non-neuronal cells expressing CXCL10 did not correlate with either the number of GrB^+^ or CD3^+^ cells (*r* = 0.46, *p* = 0.21 and *r* = 0.38, *p* = 0.30, respectively) (data not shown).

To determine if CXCL10 expression co-localized with SVV-infected neurons, adjacent ganglion sections were stained for CXCL10 transcripts and SVV ORF63 RNA by ISH. SVV ORF63 is expressed at high levels during lytic infection in vitro and in vivo but also in neurons during latency (Deitch et al. 2005; Messaoudi et al. 2009; Meyer et al. 2011; Ouwendijk et al. 2012). Most ORF63 transcripts were localized in the (peri-)nuclear region of neurons of both SVV-wt- and SVV-EGFP-infected AGMs at 9, 13, and 20 dpi (Fig. 4e). Few non-neuronal cells expressed ORF63 RNA (Fig. 4e), and more ORF63^+^ non-neuronal cells tended to be present at 9 dpi compared to 13 and 20 dpi (*p* = 0.06 and *p* = 0.09, respectively) (Fig. 4f). Whereas SVV nucleocapsid antigen was more abundant at 9 dpi compared to 13 and 20 dpi (Ouwendijk et al. 2013b), similar frequencies of SVV ORF63^+^ neurons were observed at 9 dpi (mean ± standard error of the mean, 2.8 ± 0.67 %), 13 dpi (4.5 ± 1.60 %), and 20 dpi (4.2 ± 1.0 %) (Fig. 4g). The frequency of ORF63^+^ neuron was higher at 13 dpi (*p* = 0.004) but not at 9 dpi (*p* = 0.53) in SVV-wt- compared to SVV-EGFP-infected AGMs. The number of neurons expressing ORF63 transcripts correlated significantly with the number of CXCL10^+^ neurons (*r* = 0.63, *p* < 0.001) (Fig. 4h) but not non-neuronal cells expressing CXCL10 (*r* = 0.23, *p* = 0.27) (data not shown). However, ISH analysis of adjacent sections of ganglia showed that CXCL10 and ORF63 were expressed by distinct populations of neurons, which was not different between SVV-wt-and SVV-EGFP-infected AGMs (Fig. 4i), suggesting that SVV infection did not directly induce CXCL10 expression.

### Expressions of activation and proliferation markers in ganglia

**Fig. 5:**
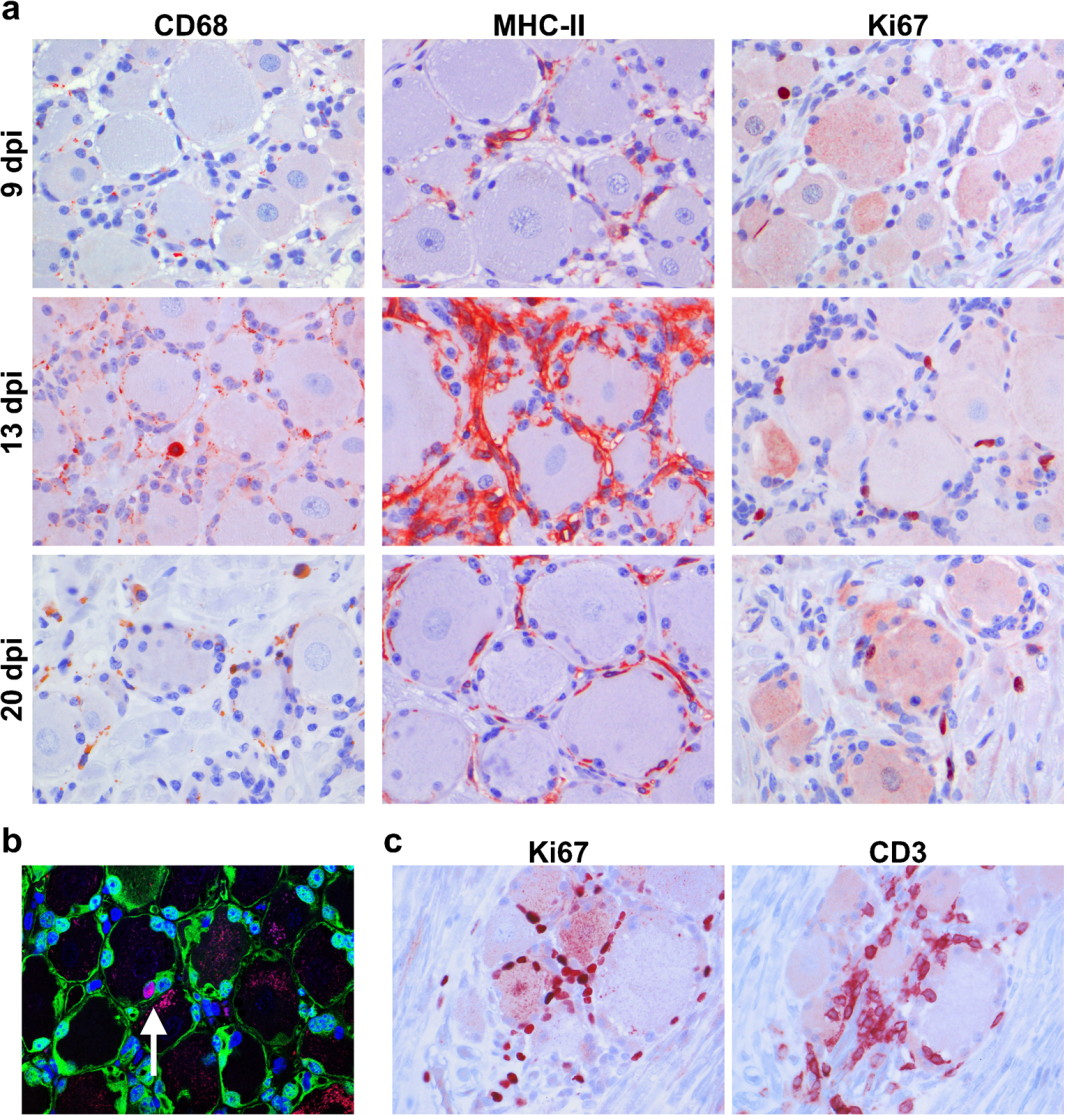
Increased expressions of activation and proliferation markers on satellite glial cells in ganglia after primary SVV infection of African green monkeys. **a** Immunohistochemical (IHC) staining of ganglia (*n* = 72 ganglia from five animals; see Table 1) at 9, 13, and 20 days post-infection (dpi) using CD68, MHC-II, and Ki67 markers. Representative IHC images are shown for 9 dpi [CD68, MHC-II, and Ki67, cervical ganglion from animal 269 (SVV-wt)], 13 dpi [CD68, sacral ganglion from animal 273 (SVV-EGFP); MHC-II and Ki67, sacral ganglion from animal 279 (SVV-wt)], and 20 dpi [CD68, MHC-II, and Ki67, sacral ganglion from animal 283 (SVV-EGFP)]. **b** Representative confocal microscopy image of a section of ganglia obtained at 13 dpi from SVV-wt-infected animal 279 stained for Ki67 (*red*) and S100B (*green*). Nuclei were counterstained with DAPI. *Arrow* indicates Ki67^+^ S100B^+^ SGC. **c** Examples of adjacent sections of sacral ganglia obtained from the SVV-EGFP-infected animal 273 at 13 dpi were stained by IHC for Ki67 and CD3. **a**, **c** Staining was visualized using 3-amino-9-ethylcarbazole as a substrate (*red*). All sections were stained in the same experiment, and nuclei were counterstained with hematoxylin (*blue*). **a**–**c** Magnification ×400

Acute herpes simplex virus (HSV) infections in mice induce activation and proliferation of SGC (Elson et al. 2003; Pereira et al. 1994). Human SGC phenotypically resemble microglia, the antigen-presenting cells of the central nervous system, which express CD68 and MHC-II molecules constitutively (Hanani 2005; van Velzen et al. 2009). Tissue sections of 72 ganglia, obtained from SVV-wt- (*n* = 2) or SVV-EGFP-infected AGMs (*n* = 3) at different dpi, were stained for the activation markers CD68 and MHC-II and for the proliferation marker Ki67. SGC expressed very low levels of CD68 and modest amounts of MHC-II at 9 dpi, while both CD68 and MHC-II were more highly expressed at 20 dpi and especially at 13 dpi compared to 9 dpi (Fig. 5a). Increased numbers of Ki67^+^ cells were detected at 13 and 20 dpi compared to 9 dpi (Fig. 5a). Notably, most Ki67^+^ were S100B^+^ SGC and infiltrating CD3^+^ T cells (Fig. 5b, c). The number and location of IHC-positive SGC and CD3^+^ T cells were not different between SVV-wt- and SVV-EGFP-infected animals (data not shown). Overall, these results suggest an active role for SGC in the ganglionic immune response following primary SVV infection.

## Discussion

The role of the immune response in ganglia after varicella is largely unknown. Ganglia obtained from varicella patients show limited histopathological changes, suggesting control of virus replication (Berg et al. 1969; Cheatham et al. 1956; Nagashima et al. 1975). Primary SVV infection induces an analogous minor histopathology and mild inflammation in ganglia of AGM (Ouwendijk et al. 2013b). Our current study aim to further characterize the immune response in ganglia after acute SVV infection reports two main findings. Primary SVV infection induces (1) a transient increase in CD8^dim^ and CD8^bright^ memory T cells and (2) local expression of the chemokine CXCL10 and activation/proliferation of both resident neuron-interacting SGC and infiltrating T cells in ganglia.

SVV enters ganglia at 6 dpi, ganglionic viral DNA loads peak at 9 dpi, and SVV latency is probably established from 3 to 10 weeks after infection (Mahalingam et al. 2001; Messaoudi et al. 2009; Ouwendijk et al. 2012, 2013b). The significantly higher SVV DNA load and more abundant SVV nucleocapsid antigen expression in ganglia at 9 dpi compared to 13 and 20 dpi (Ouwendijk et al. 2013b) suggest an initial phase of virus replication followed by establishment of latency. This is supported by the detection immediate-early, early, and late transcripts in ganglia during acute SVV infection (Nagel et al. 2013; Ou et al. 2007), which contrasts the restricted viral gene expression detected in SVV latently infected ganglia (Messaoudi et al. 2009; Ouwendijk et al. 2012). The decline in SVV DNA load in ganglia parallels the recovery from skin rash and emergence of systemic humoral and cell-mediated anti-SVV responses (Haberthur et al. 2011; Messaoudi et al. 2009; Ouwendijk et al. 2012). Herein, we show that a reduction in viral load in ganglia at 9 to 13 dpi (Ouwendijk et al. 2013b) (Table 1) is accompanied by an influx of CD8^dim^ and CD8^bright^ memory T cells. Specific enrichment of CD8^dim^ T cells, which develop from CD4^+^ T cells and recognize antigen presented by MHC-II molecules (Beaumier et al. 2009), and increased MHC-II expression on SGC suggest a critical role for MHC-II-restricted T cell responses. Indeed, depletion of CD4^+^ T cells, but not CD8^+^ T cells or B cells, during primary SVV infection resulted in sustained lytic viral gene expression in ganglia (Meyer et al. 2013). Overall, the data suggest that T cells infiltrating ganglia contribute to the control of SVV replication and potentially to the establishment of latency.

The increased number of T cells in ganglia after primary SVV infection might reflect recruitment of T cells via expression of chemokines such as CXCL10, followed by local proliferation of T cells. Limited numbers of Ki67^+^-proliferating T cells were detected in ganglia at 13 and 20 dpi. T cell proliferation might be induced by infiltrating CD11c^+^ myeloid cells or activated SGC (Wakim et al. 2008). Additionally, we show that primary SVV infection induces CXCL10 expression in neurons, SGC, and infiltrating lymphocytes. The chemokine CXCL10 (interferon-inducible protein-10) is secreted by a variety of cell types in response to type I and II interferons and orchestrates migration of CXCR3-bearing activated memory T cells and natural killer cells into tissues (Loetscher et al. 1996; Luster et al. 1985; Sallusto et al. 1998; Zlotnik and Yoshie 2012). Previous studies suggest the prime role of neuronal CXCL10 production in recruitment of T cells in response to neurotropic viral infection such as rabies virus and West Nile virus (Chai et al. 2015; Klein et al. 2005). Similarly, the current study showed that neuronal CXCL10 expression correlated significantly with activated GrB^+^ T cells infiltrating ganglia and to a limited extent with the total number of T cells. Whereas SVV-specific T cells are already detected in blood at 7 dpi (Haberthur et al. 2011; Messaoudi et al. 2009; Ouwendijk et al. 2012), additional studies are warranted to demonstrate the antigen specificity of T cells in ganglia after primary SVV infection.

Neuronal CXCL10 expression is associated with acute virus replication in ganglia, while (infiltrating) non-neuronal cells may produce CXCL10 for extended periods after cessation of virus replication (Ouwendijk et al. 2013a; Steain et al. 2011; Theil et al. 2003). Our demonstration of induction of CXCL10 expression in neurons after primary SVV infection is similar to earlier observations of active VZV replication, both in ganglia explants infected with VZV in vitro and in ganglia obtained at autopsy from zoster patients (Steain et al. 2011). In contrast, CXCL10 transcripts were found only in non-neuronal cells in ganglia >1 week after SVV reactivation and in HSV-1 latently infected human trigeminal ganglia (Ouwendijk et al. 2013a; Theil et al. 2003). Herein, we showed that SVV ORF63 transcripts correlated with CXCL10 RNA in neurons. However, neither neuronal nor non-neuronal CXCL10 expression co-localized with SVV-infected neurons, suggesting that SVV infection impaired CXCL10 expression or, more likely, that SVV-infected neurons and/or infiltrating lymphocytes secrete CXCL10-inducing factors. Potent inducers of CXCL10 include type I and II interferons that signal via signal transducers and activators of transcription 1 (STAT1) (Loetscher et al. 1996; Luster et al. 1985; Zlotnik and Yoshie 2012). However, only rare neurons, SGC, and infiltrating cells stained for nuclear phosphorylated STAT1 (data not shown), suggesting that other cytokines or chemokines are involved (Dong et al. 2013).

Innate ganglionic immune responses are sufficient to control virus replication and establishment of VZV latency in an experimental chimeric mouse model of VZV infection (Zerboni et al. 2005). SGC can produce inflammatory mediators, such as prostaglandins, tumor necrosis factor (TNF)-α, and interleukin (IL)-6 and IL-15, and can modulate local T cell function (Hanani 2005; van Velzen et al. 2009). HSV-1 infection of mice increases MHC-I and MHC-II expressions on SGC, causes SGC proliferation, and induces TNF-α and IL-6 production in SGC surrounding HSV-infected neurons (Elson et al. 2003; Mori et al. 2003; Pereira et al. 1994; Shimeld et al. 1999). Similarly, we find that primary SVV infection causes increased CD68 and MHC-II expressions on SGC and, possibly, restricted SGC proliferation in ganglia along the entire neuraxis. Together, the data suggest an active role for SGC in the ganglionic immune response following alphaherpesvirus infection.

SVV-EGFP was attenuated compared to SVV-wt (Ouwendijk et al. 2013b), as shown by the reduced viral DNA load in ganglia and lower number of neurons expressing SVV ORF63 transcripts in SVV-EGFP- compared to SVV-wt-infected AGMs (Table 1 and Fig. 4b). Nevertheless, ganglia obtained from both SVV-wt- and SVV-EGFP-infected AGMs showed broad activation of SGC and increased numbers of CD3^+^ T cells and CD11c^+^ myeloid cells. By contrast, neuronal CXCL10 expression and the associated number of GrB^+^ T cells (Fig. 4c) were significantly increased in SVV-wt-infected AGM, consistent with the observed correlation between SVV ORF63 RNA and CXCL10 transcript expression in neurons (Fig. 4g). Collectively, the data suggest that primary SVV infection induces a similar number of immune infiltrates independent of viral load and the SVV virus (SVV-wt and SVV-EGFP) used. Notably, the expressions of immune effector molecules (granzyme B and CXCL10) did correlate with viral load.

In conclusion, the emergence of local immune responses coincided with the decline in viral DNA load in ganglia, suggesting that intra-ganglionic immunity contributes to restricting local SVV infection. These data implicate a multifaceted immune response in ganglia after primary SVV infection involving neurons, SGC, and infiltrating CD11c^+^ myeloid cells and T cells. Further studies in larger cohorts are needed to conclusively define the cell types and mediators that control virus replication in ganglia and establishment of SVV latency.

## References

[CR1] Arvin AM, Gilden D, Knipe DM, Howley PM (2013). Varicella-zoster virus. Fields virology.

[CR2] Arvin AM, Koropchak CM, Williams BR, Grumet FC, Foung SK (1986). Early immune response in healthy and immunocompromised subjects with primary varicella-zoster virus infection. J Infect Dis.

[CR3] Beaumier CM, Harris LD, Goldstein S, Klatt NR, Whitted S, McGinty J, Apetrei C, Pandrea I, Hirsch VM, Brenchley JM (2009). CD4 downregulation by memory CD4+ T cells in vivo renders African green monkeys resistant to progressive SIVagm infection. Nat Med.

[CR4] Berg R, Hansson O, Nordbring F, Sourander P, Stenram U, Willen R (1969). Fatal varicella generalisata in a child with immunopathy and hereditary neurological syndrome. A report with autopsy, electron microscopy and virus isolation. Virchows Arch A Pathol Pathol Anat.

[CR5] Chai Q, She R, Huang Y, Fu ZF (2015). Expression of neuronal CXCL10 induced by rabies virus infection initiates infiltration of inflammatory cells, production of chemokines and cytokines, and enhancement of blood-brain barrier permeability. J Virol.

[CR6] Cheatham WJ, Dolan TF, Dower JC, Weller TH (1956). Varicella: report of two fatal cases with necropsy, virus isolation, and serologic studies. Am J Pathol.

[CR7] Deitch SB, Gilden DH, Wellish M, Smith J, Cohrs RJ, Mahalingam R (2005). Array analysis of simian varicella virus gene transcription in productively infected cells in tissue culture. J Virol.

[CR8] Dong S, Zhang X, He Y, Xu F, Li D, Xu W, Wang H, Yin Y, Cao J (2013). Synergy of IL-27 and TNF-alpha in regulating CXCL10 expression in lung fibroblasts. Am J Respir Cell Mol Biol.

[CR9] Dueland AN, Martin JR, Devlin ME, Wellish M, Mahalingam R, Cohrs R, Soike KF, Gilden DH (1992). Acute simian varicella infection. Clinical, laboratory, pathologic, and virologic features. Lab Invest.

[CR10] Elson K, Speck P, Simmons A (2003). Herpes simplex virus infection of murine sensory ganglia induces proliferation of neuronal satellite cells. J Gen Virol.

[CR11] Gowrishankar K, Steain M, Cunningham AL, Rodriguez M, Blumbergs P, Slobedman B, Abendroth A (2010). Characterization of the host immune response in human ganglia after herpes zoster. J Virol.

[CR12] Haberthur K, Engelmann F, Park B, Barron A, Legasse A, Dewane J, Fischer M, Kerns A, Brown M, Messaoudi I (2011). CD4 T cell immunity is critical for the control of simian varicella virus infection in a nonhuman primate model of VZV infection. PLoS Pathog.

[CR13] Hanani M (2005). Satellite glial cells in sensory ganglia: from form to function. Brain Res Brain Res Rev.

[CR14] Kennedy PG, Grinfeld E, Traina-Dorge V, Gilden DH, Mahalingam R (2004). Neuronal localization of simian varicella virus DNA in ganglia of naturally infected African green monkeys. Virus Genes.

[CR15] Klein RS, Lin E, Zhang B, Luster AD, Tollett J, Samuel MA, Engle M, Diamond MS (2005). Neuronal CXCL10 directs CD8+ T-cell recruitment and control of West Nile virus encephalitis. J Virol.

[CR16] Kolappaswamy K, Mahalingam R, Traina-Dorge V, Shipley ST, Gilden DH, Kleinschmidt-Demasters BK, McLeod CG, Hungerford LL, DeTolla LJ (2007). Disseminated simian varicella virus infection in an irradiated rhesus macaque (Macaca mulatta). J Virol.

[CR17] Loetscher M, Gerber B, Loetscher P, Jones SA, Piali L, Clark-Lewis I, Baggiolini M, Moser B (1996). Chemokine receptor specific for IP10 and mig: structure, function, and expression in activated T-lymphocytes. J Exp Med.

[CR18] Luster AD, Unkeless JC, Ravetch JV (1985). Gamma-interferon transcriptionally regulates an early-response gene containing homology to platelet proteins. Nature.

[CR19] Mahalingam R, Gilden DH, Arvin A, Campadelli-Fiume G, Mocarski E, Moore PS, Roizman B, Whitley R, Yamanishi K (2007). Simian varicella virus. Human herpesviruses: biology, therapy, and immunoprophylaxis.

[CR20] Mahalingam R, Smith D, Wellish M, Wolf W, Dueland AN, Cohrs R, Soike K, Gilden D (1991). Simian varicella virus DNA in dorsal root ganglia. Proc Natl Acad Sci U S A.

[CR21] Mahalingam R, Wellish M, White T, Soike K, Cohrs R, Kleinschmidt-DeMasters BK, Gilden DH (1998). Infectious simian varicella virus expressing the green fluorescent protein. J Neurovirol.

[CR22] Mahalingam R, Wellish M, Soike K, White T, Kleinschmidt-DeMasters BK, Gilden DH (2001). Simian varicella virus infects ganglia before rash in experimentally infected monkeys. Virology.

[CR23] Mahalingam R, Traina-Dorge V, Wellish M, Lorino R, Sanford R, Ribka EP, Alleman SJ, Brazeau E, Gilden DH (2007). Simian varicella virus reactivation in cynomolgus monkeys. Virology.

[CR24] Malavige GN, Jones L, Kamaladasa SD, Wijewickrama A, Seneviratne SL, Black AP, Ogg GS (2008). Viral load, clinical disease severity and cellular immune responses in primary varicella zoster virus infection in Sri Lanka. PLoS One.

[CR25] Messaoudi I, Barron A, Wellish M, Engelmann F, Legasse A, Planer S, Gilden D, Nikolich-Zugich J, Mahalingam R (2009). Simian varicella virus infection of rhesus macaques recapitulates essential features of varicella zoster virus infection in humans. PLoS Pathog.

[CR26] Meyer C, Kerns A, Barron A, Kreklywich C, Streblow DN, Messaoudi I (2011). Simian varicella virus gene expression during acute and latent infection of rhesus macaques. J Neurovirol.

[CR27] Meyer C, Dewane J, Kerns A, Haberthur K, Barron A, Park B, Messaoudi I (2013). Age and immune status of rhesus macaques impact simian varicella virus gene expression in sensory ganglia. J Virol.

[CR28] Mori I, Goshima F, Koshizuka T, Imai Y, Kohsaka S, Koide N, Sugiyama T, Yoshida T, Yokochi T, Kimura Y, Nishiyama Y (2003). Iba1-expressing microglia respond to herpes simplex virus infection in the mouse trigeminal ganglion. Brain Res Mol Brain Res.

[CR29] Nagashima K, Nakazawa M, Endo H (1975). Pathology of the human spinal ganglia in varicella-zoster virus infection. Acta Neuropathol.

[CR30] Nagel MA, Choe A, Gilden D, Traina-Dorge V, Cohrs RJ, Mahalingam R (2013). GeXPS multiplex PCR analysis of the simian varicella virus transcriptome in productively infected cells in culture and acutely infected ganglia. J Virol Methods.

[CR31] Ou Y, Davis KA, Traina-Dorge V, Gray WL (2007). Simian varicella virus expresses a latency-associated transcript that is antisense to open reading frame 61 (ICP0) mRNA in neural ganglia of latently infected monkeys. J Virol.

[CR32] Ouwendijk WJ, Verjans GM (2015). Pathogenesis of varicelloviruses in primates. J Pathol.

[CR33] Ouwendijk WJ, Mahalingam R, Traina-Dorge V, van Amerongen G, Wellish M, Osterhaus AD, Gilden D, Verjans GM (2012). Simian varicella virus infection of Chinese rhesus macaques produces ganglionic infection in the absence of rash. J Neurovirol.

[CR34] Ouwendijk WJ, Abendroth A, Traina-Dorge V, Getu S, Steain M, Wellish M, Andeweg AC, Osterhaus AD, Gilden D, Verjans GM, Mahalingam R (2013). T-cell infiltration correlates with CXCL10 expression in ganglia of cynomolgus macaques with reactivated simian varicella virus. J Virol.

[CR35] Ouwendijk WJ, Mahalingam R, de Swart RL, Haagmans BL, van Amerongen G, Getu S, Gilden D, Osterhaus AD, Verjans GM (2013). T-cell tropism of simian varicella virus during primary infection. PLoS Pathog.

[CR36] Pereira RA, Tscharke DC, Simmons A (1994). Upregulation of class I major histocompatibility complex gene expression in primary sensory neurons, satellite cells, and Schwann cells of mice in response to acute but not latent herpes simplex virus infection in vivo. J Exp Med.

[CR37] Picker LJ, Terstappen LW, Rott LS, Streeter PR, Stein H, Butcher EC (1990). Differential expression of homing-associated adhesion molecules by T cell subsets in man. J Immunol.

[CR38] Pitcher CJ, Hagen SI, Walker JM, Lum R, Mitchell BL, Maino VC, Axthelm MK, Picker LJ (2002). Development and homeostasis of T cell memory in rhesus macaque. J Immunol.

[CR39] Sallusto F, Lenig D, Mackay CR, Lanzavecchia A (1998). Flexible programs of chemokine receptor expression on human polarized T helper 1 and 2 lymphocytes. J Exp Med.

[CR40] Shimeld C, Easty DL, Hill TJ (1999). Reactivation of herpes simplex virus type 1 in the mouse trigeminal ganglion: an in vivo study of virus antigen and cytokines. J Virol.

[CR41] Steain M, Gowrishankar K, Rodriguez M, Slobedman B, Abendroth A (2011). Upregulation of CXCL10 in human dorsal root ganglia during experimental and natural varicella-zoster virus infection. J Virol.

[CR42] Steain M, Sutherland JP, Rodriguez M, Cunningham AL, Slobedman B, Abendroth A (2014). Analysis of T cell responses during active varicella-zoster virus reactivation in human ganglia. J Virol.

[CR43] Theil D, Derfuss T, Paripovic I, Herberger S, Meinl E, Schueler O, Strupp M, Arbusow V, Brandt T (2003). Latent herpesvirus infection in human trigeminal ganglia causes chronic immune response. Am J Pathol.

[CR44] van Velzen M, Laman JD, Kleinjan A, Poot A, Osterhaus AD, Verjans GM (2009). Neuron-interacting satellite glial cells in human trigeminal ganglia have an APC phenotype. J Immunol.

[CR45] Verjans GM, Hintzen RQ, van Dun JM, Poot A, Milikan JC, Laman JD, Langerak AW, Kinchington PR, Osterhaus AD (2007). Selective retention of herpes simplex virus-specific T cells in latently infected human trigeminal ganglia. Proc Natl Acad Sci U S A.

[CR46] Vukmanovic-Stejic M, Sandhu D, Sobande TO, Agius E, Lacy KE, Riddell N, Montez S, Dintwe OB, Scriba TJ, Breuer J, Nikolich-Zugich J, Ogg G, Rustin MH, Akbar AN (2013). Varicella zoster-specific CD4 + Foxp3+ T cells accumulate after cutaneous antigen challenge in humans. J Immunol.

[CR47] Vukmanovic-Stejic M, Sandhu D, Seidel JA, Patel N, Sobande TO, Agius E, Jackson SE, Fuentes-Duculan J, Suarez-Farinas M, Mabbott NA, Lacy KE, Ogg G, Nestle FO, Krueger JG, Rustin MH, Akbar AN (2015). The characterization of varicella zoster virus-specific T cells in skin and blood during aging. J Invest Dermatol.

[CR48] Wakim LM, Waithman J, van Rooijen N, Heath WR, Carbone FR (2008). Dendritic cell-induced memory T cell activation in nonlymphoid tissues. Science.

[CR49] Weinberg A, Zhang JH, Oxman MN, Johnson GR, Hayward AR, Caulfield MJ, Irwin MR, Clair J, Smith JG, Stanley H, Marchese RD, Harbecke R, Williams HM, Chan IS, Arbeit RD, Gershon AA, Schodel F, Morrison VA, Kauffman CA, Straus SE, Schmader KE, Davis LE, Levin MJ, Investigators USDoVACSPSPS (2009). Varicella-zoster virus-specific immune responses to herpes zoster in elderly participants in a trial of a clinically effective zoster vaccine. J Infect Dis.

[CR50] Zak-Prelich M, McKenzie RC, Sysa-Jedrzejowska A, Norval M (2003). Local immune responses and systemic cytokine responses in zoster: relationship to the development of postherpetic neuralgia. Clin Exp Immunol.

[CR51] Zerboni L, Ku CC, Jones CD, Zehnder JL, Arvin AM (2005). Varicella-zoster virus infection of human dorsal root ganglia in vivo. Proc Natl Acad Sci U S A.

[CR52] Zlotnik A, Yoshie O (2012). The chemokine superfamily revisited. Immunity.

